# Type 1 diabetes management: Room for improvement

**DOI:** 10.1111/1753-0407.13368

**Published:** 2023-02-17

**Authors:** Rita D. M. Varkevisser, Erwin Birnie, Dick Mul, Peter R. van Dijk, Henk‐Jan Aanstoot, Bruce H. R. Wolffenbuttel, Melanie M. van der Klauw

**Affiliations:** ^1^ Department of Endocrinology University of Groningen, University Medical Center Groningen Groningen The Netherlands; ^2^ Diabeter Nederland, Center for Focussed Diabetes Care and Research Rotterdam The Netherlands; ^3^ Department of Genetics University of Groningen, University Medical Center Groningen Groningen The Netherlands

**Keywords:** blood glucose, blood pressure, cardiovascular diseases, cholesterol, LDL, diabetes mellitus type 1, lipid metabolism, 1型糖尿病, 心血管疾病, 血糖, 血压, 脂代谢, 胆固醇, LDL。

## Abstract

**Aims/Hypothesis:**

Optimal diabetes care and risk factor management are important to delay micro‐ and macrovascular complications in individuals with type 1 diabetes (T1D). Ongoing improvement of management strategies requires the evaluation of target achievement and identification of risk factors in individuals who do (or do not) achieve these targets.

**Methods:**

Cross‐sectional data were collected from adults with T1D visiting six diabetes centers in the Netherlands in 2018. Targets were defined as glycated hemoglobin (HbA1c) <53 mmol/mol, low‐density lipoprotein‐cholesterol (LDL‐c) <2.6 mmoL/L (no cardiovascular disease [CVD] present) or <1.8 mmoL/L (CVD present), or blood pressure (BP) <140/90 mm Hg. Target achievement was compared for individuals with and without CVD.

**Results:**

Data from 1737 individuals were included. Mean HbA1c was 63 mmol/mol (7.9%), LDL‐c was 2.67 mmoL/L, and BP 131/76 mm Hg. In individuals with CVD, 24%, 33%, and 46% achieved HbA1c, LDL‐c, and BP targets respectively. In individuals without CVD these percentages were 29%, 54%, and 77%, respectively. Individuals with CVD did not have any significant risk factors for HbA1c, LDL‐c, and BP target achievement. In comparison, individuals without CVD were more likely to achieve glycemic targets if they were men and insulin pump users. Smoking, microvascular complications, and the prescription of lipid‐lowering and antihypertensive medication were negatively associated with glycemic target achievement. No characteristics were associated with LDL‐c target achievement. Microvascular complications and antihypertensive medication prescription were negatively associated with BP target attainment.

**Conclusion:**

Opportunities for improvement of diabetes management exist for the achievement of glycemic, lipid, and BP targets but may differ between individuals with and without CVD.

## Graphical abstract

**Highlights**

- Achievement of glycemic, lipid, and blood pressure targets are suboptimal.
- Individuals with type 1 diabetes and cardiovascular disease (CVD) have more difficulty reaching treatment targets than individuals with diabetes without CVD.
- More consideration may be required for individuals with a previous cardiovascular event and type 1 diabetes.

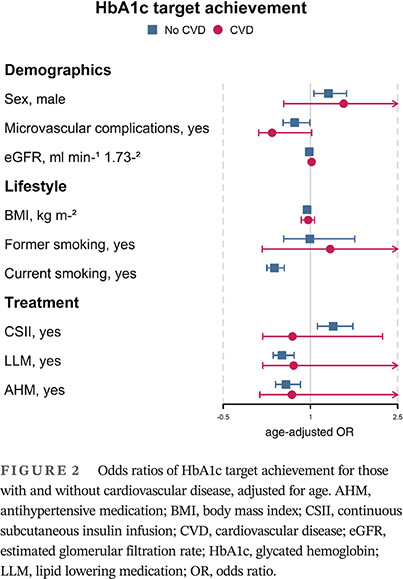

## 摘要

**目的:** 最佳的糖尿病护理和危险因素管理对于延缓1型糖尿病(T1D)患者的微血管和大血管并发症非常重要。管理战略的持续改进需要对目标实现情况进行评估, 并确定实现(或未实现)这些目标的个人风险因素。

**方法:** 于2018年收集了荷兰六个糖尿病中心成年T1D患者的横断面数据。目标定义为HbA_1c_ <53mmol/mol、低密度脂蛋白胆固醇(LDL‐C)<2.6mmol/l(无心血管疾病(CVD)存在)或<1.8mmol/l(存在CVD), 或血压(BP)<140/90mmHg。比较了有无CVD的个体的目标达成情况。

**结果:** 纳入了1737人的数据。平均HbA_1c_为63 mmol/mol (7.9%), LDL‐C为2.67 mmol/l, BP 为131/76 mmHg。在CVD患者中, 分别有24%、33%和46%达到了HbA_1c_、LDL‐C和BP目标。在没有心血管疾病的个体中, 这些比例分别为29%、54%和77%。CVD患者在HbA_1c_、LDL‐C和BP目标实现方面没有任何显著的危险因素。相比之下, 没有心血管疾病的人更有可能达到血糖目标, 如果他们是男性和胰岛素泵使用者。吸烟、微血管并发症、降脂处方和降压药与实现血糖目标呈负相关。没有与LDL‐C目标实现相关的特征。微血管并发症和抗高血压药物处方与血压达标呈负相关。

**结论:** 改善糖尿病管理的机会存在于实现血糖、血脂和血压的达标, 但可能在有和没有CVD的个体之间有所不同。

## 1 INTRODUCTION

Individuals with type 1 diabetes (T1D) are at risk for morbidity and mortality as a result of diabetes‐related complications.^1^ Although there has been an overall improvement in life expectancy and quality of life over the years, there is still a discrepancy of 11–13 years in life expectancy in individuals with T1D in comparison to controls without diabetes.^2^ This reduction in life expectancy remains largely attributed to cardiovascular disease (CVD).^2^ The Diabetes Control and Complications Trial/Epidemiology of Diabetes Interventions and Complications trial (DCCT/EDIC) unequivocally demonstrated the importance of strict glycemic control to prevent macrovascular complications.^3^ Although glycemic control is the cornerstone for T1D management, studies have shown that both lipid and blood pressure levels also affect the development of micro‐ and macrovascular complications.^1^, ^4^ Moreover, dyslipidemia and hypertension have been hypothesized to have synergistic effects in cardiovascular risk.^5^ Lowering blood pressure, and to a lesser extent low‐density lipoprotein‐cholesterol (LDL‐c), has been shown to have protective effects against CVD in individuals with T1D.^6^, ^7^

Assessing whether individuals are reaching treatment goals and identifying subgroup differences in reaching these targets is necessary to improve patient care and self‐management. Despite the large burden of CVD in individuals with T1D, reports on the achievement of LDL‐c and blood pressure targets are limited. In addition, percentages of individuals with T1D reaching treatment targets^6^, ^8^ in glycated hemoglobin (HbA1c), lipid, and blood pressure achievement vary from 10%–39%, 24%–73%, to 62%–84%, respectively.^6^, ^9^ Importantly, assessing the subgroup of individuals with T1D who already experienced a cardiovascular event is particularly relevant, as these individuals are still at (high) risk for recurrent CVD.^10^ Data on target achievement in this group are extremely limited.

In this study, we assess the percentage of adults with T1D who achieve HbA1c, LDL‐c, and blood pressure targets, with and without known CVD across six diabetes centers in the Netherlands. Furthermore, differences between those who achieve or do not achieve targets are described to find potential subgroups that require more, or different, attention for cardiovascular risk management.

## 2 RESEARCH DESIGN AND METHODS

### 2.1 Study design and population

This is a cross‐sectional registry‐based study. Electronic patient data were collected from two centers: Diabeter, a specialized T1D treatment and research center with five locations throughout the Netherlands, and the University Medical Center Groningen (UMCG), the Netherlands. Individuals visiting these clinics between 1 January 2018 and 31 December 2018 were included if they were over the age of 18, were diagnosed with T1D, and had used insulin for at least 1 year. T1D diagnosis was determined by the presence of American Diabetes Association (ADA) criteria for diabetes mellitus and a clinical presentation typical for T1D or the presence of autoantibodies.^11^ Individuals were excluded if no measurements were present for LDL‐c, blood pressure, or HbA1c levels in 2018.

The Medical Ethical Review Board of the UMCG, Groningen, the Netherlands, declared that this study was not subject to the Dutch Medical Research Involving Human Subjects Act (WMO) and a waiver was granted. The institutional review board approved the study protocol (202000883).

Data extraction for this study is described in detail elsewhere.^12^ In summary, demographic, anthropometric, laboratory, and medication data were extracted from electronic medical records (EMRs) from the subjects' annual diabetes complication screening visits in 2018.

### 2.2 Variable definitions

Demographic data included age and diabetes duration, sex, and ethnicity or parental country of birth. Ethnicity was classified as either western European or non‐western European, as no meaningful subgroups could be formed in the latter. When ethnicity was not available, parental country of birth was used to determine ethnicity. If at least one parent was born outside of western Europe, the individual was considered non‐western European.

CVD was defined as the presence of a positive medical history for coronary artery disease, cerebrovascular disease or transient ischemic attack, peripheral arterial disease, or the prescription of platelet aggregation inhibitors. An individual was considered to have microvascular complications if they had a positive medical history for retinopathy, neuropathy, or nephropathy.

Anthropometric data on blood pressure, height, and weight were also extracted. Body mass index (BMI) was calculated by dividing weight in kilograms by the height in meters squared.

Laboratory measurements included HbA1c, serum creatinine, and LDL‐c. Estimated glomerular filtration rate (eGFR) was calculated using the Chronic Kidney Disease Epidemiology Collaboration formula.^13^

Medication prescribed was extracted from the EMRs and relevant medication was coded based on their mechanism of action: antihypertensive, lipid lowering, and platelet aggregation inhibitors. For antihypertensive medication, the number of antihypertensive medications prescribed was calculated and classified as either none, one, two, or three or more.

### 2.3 Achievement of targets

The achievement of targets was assessed for the outcomes glycemic control, LDL‐c, and blood pressure. The ADA guidelines recommend the HbA1c target to be <53 mmol/mol (7.0%), unless stricter targets can be achieved without risk of hypoglycemia (HbA1c <48 mmol/mol [6.5%]) or when an individual's life expectancy is limited (HbA1c <64 mmol/mol [8.0%]), or the harms outweigh the benefits.^14^ The Dutch guidelines similarly recommend striving for a HbA1c <53 mmol/mol, unless the individual is over the age of 70 and has had T1D for longer than 10 years, in which case a higher limit is accepted (HbA1c <64 mmol/mol).^15^ Because the distinction as to when to choose a goal of <48 or <64 mmol/mol is difficult to make with the available data, the HbA1c target of <53 mmol/mol was used, which is comparable to other studies.^9^

LDL‐c targets were <2.6 mmoL/L for those without CVD and <1.8 mmoL/L for those with CVD.^16^ The Dutch guidelines recommend a LDL‐c <2.6 mmoL/L in individuals with high risk of CVD morbidity and mortality.^16^ Although the Dutch guidelines do not recommend lipid‐lowering medication in individuals <40 years with a low systematic coronary risk evaluation (SCORE),^16^ treatment may be considered.^17^

Blood pressure targets were achieved if the measured blood pressure in the outpatient clinic setting was <140/90 mm Hg.^16^ Although individuals with nephropathy would be considered for stricter targets such as <130/80 mm Hg, for the purpose of macrovascular complication risk reduction we used the target of <140/90 mm Hg.

### 2.4 Statistical analysis

All statistical analyses were conducted using R Statistical software,^18^ R Studio Software,^19^ and R packages.^20^, ^21^, ^22^ The study population is described and presented as unadjusted means with SDs, median with interquartile range, or counts with percentages.

Differences in the characteristics between those with and without CVD were evaluated with unpaired *t* tests, Wilcoxon rank‐sum, chi‐square, or Fisher exact tests where appropriate.

The achievement of targets was further analyzed for those without CVD and those with CVD. Percentages of individuals achieving the target were calculated for each target separately for those with and without CVD.

For the CVD positive (CVD+) and CVD negative (CVD−) group, age‐adjusted odds ratios (aORs) were calculated using multiple logistic regression analysis to determine which risk factors may have an impact on target achievement. Adjustment was made for age as age is a confounder for the achievement of HbA1c, LDL‐c, and blood pressure.^23^ aORs were calculated for the following risk factors: sex, continuous subcutaneous insulin infusion (CSII), BMI, microvascular complications, smoking, eGFR, lipid‐lowering medication (LLM), and antihypertensive medication (AHM). Diabetes duration was excluded as diabetes duration and age were highly correlated.

## 3 RESULTS

A total of 2293 individuals visited the six diabetes centers for annual diabetes screening. After excluding individuals with missing data for HbA1c, LDL‐c, and blood pressure, 1737 were included in this study. Table 1 shows the characteristics of the study population by CVD status.

**TABLE 1:** Study population characteristics, and differences in those with and without cardiovascular disease (CVD).

Characteristics	Whole population (*n* = 1737)	CVD‐ (*n* = 1650)	CVD+ (n = 87)
Age, years	27 (22, 43)	26 (22, 39)	61 (53, 67)
Sex, *n* (%) women	876 (50)	840 (51)	36 (41)
Ethnicity, *n* (%) Western European	1639 (94)	1553 (94)	86 (99)
Diabetes duration, years	16 (10, 24)	15 (10, 22)	40 (33, 50)
CSII, *n* (%) yes	917 (53)	892 (55)	25 (29)
BMI, kg/m^2^	25.6 ± 4.4	25.5 ± 4.4	27.1 ± 5.0
Systolic blood pressure, mm Hg	131 ± 13	130 ± 13	141 ± 18
Smoking, *n* (%) yes			
Current smoker count, *n* (%)	234 (14)	216 (14)	18 (21)
Former smoker count, *n* (%)	61 (3.7)	54 (3.5)	7 (8.1)
Never smoker count, *n* (%)	1355 (82)	1294 (83)	61 (71)
HbA1c, mmol/mol	63 ± 16	63 ± 16	63 ± 13
HbA1c, %	7.9 ± 1.5	7.9 ± 1.5	7.9 ± 1.2
eGFR, ml min¯^1^ 1.73¯^2^	98 (82, 117)	100 (84, 118)	63 (53, 80)
Albumin creatinine ratio, mg/mmol	0.9 (0.50, 1.91)	0.83 (0.50, 1.83)	1.70 (0.78, 7.55)
LDL‐cholesterol, mmol/L	2.67 ± 0.79	2.69 ± 0.78	2.33 ± 0.90
Microvascular complications, *n* (%) yes	369 (21)	310 (19)	59 (68)
Lipid‐lowering medication, *n* (%) yes	357 (21)	281 (17)	76 (87)
Antihypertensive medication, *n* (%) yes	303 (17)	224 (14)	79 (91)
Platelet aggregation inhibitors, *n* (%) yes	60 (35)	12 (7.3)	48 (55)
Coronary heart disease, *n* (%) yes	57 (3.3)	‐	57 (65)
Cerebral vascular accident or transient ischemic attack, *n* (%) yes	13 (0.8)	‐	13 (15)
Peripheral arterial disease, *n* (%) yes	32 (1.8)	‐	32 (37)

*Note*: Data are presented as means ± SD, medians (quartile 1, quartile 3), and *n* (%). Missing data. *N* = 1; microvascular complications, cardiovascular disease. *N* = 5; CSII. *N* = 73; BMI. *N* = 87; smoking. *N* = 1097; albumin creatinine ratio.

Abbreviations: BMI, body mass index; CSII, continuous subcutaneous insulin infusion; eGFR, estimated glomerular filtration rate; HbA1c, glycated hemoglobin; LDL, low‐density lipoprotein.

Participants with CVD were significantly older, had a longer diabetes duration, a greater BMI and higher systolic blood pressure, and were more often smokers or former smokers in comparison to the CVD‐ group. The percentage of individuals with microvascular complications, and with any medication prescription ‐‐ for all working mechanisms ‐‐ was significantly greater in individuals with CVD, whereas CSII use was significantly lower among those with CVD.

### 3.1 Achievement of targets

**FIGURE 1:**
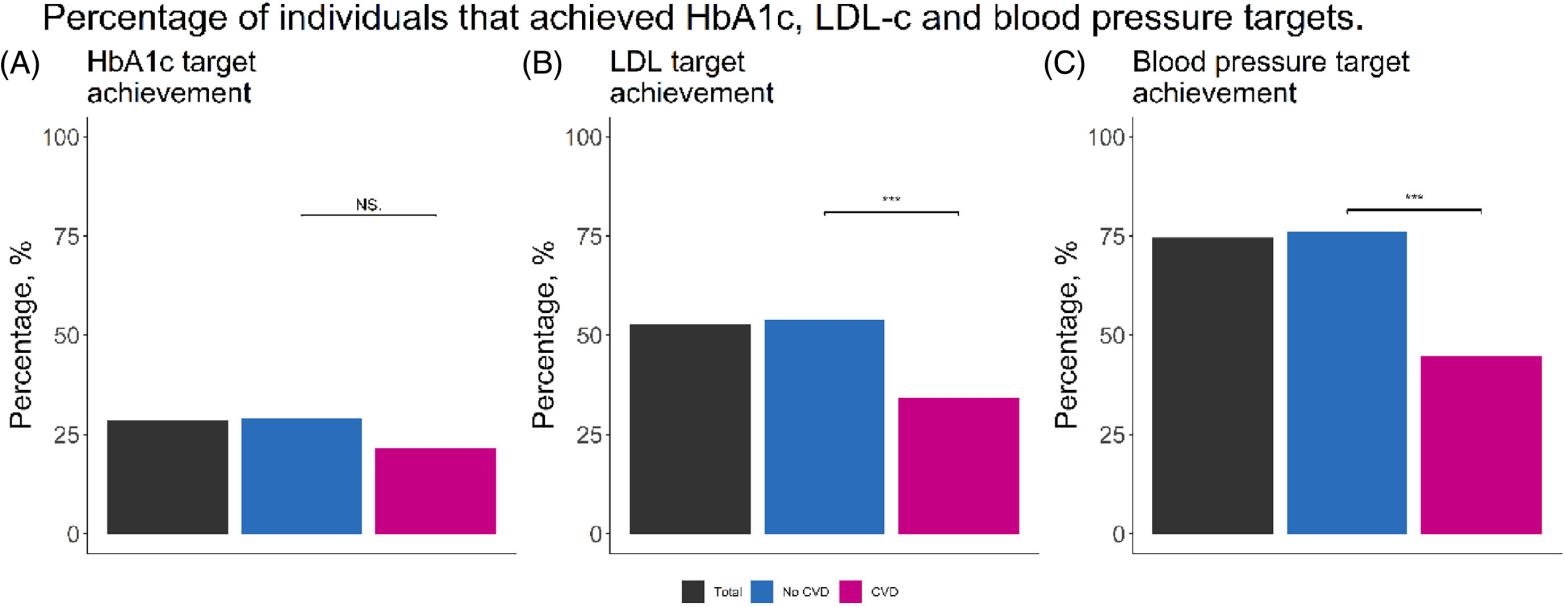
Distribution of targets and percentage of targets achieved for glycated hemoglobin (HbA1c), LDL‐cholesterol (LDL‐c), and systolic blood pressure. In blue the no cardiovascular disease (CVD) group, in pink the CVD group, and in black the total group are shown. (A) percentage of individuals achieving the target HbA1c of <53 mmol/mol (7.0%), (B) percentage of individuals achieving target LDL‐c of <1.8 mmoL/L (CVD) and <2.6 mmoL/L (no CVD), (f) percentage of individuals achieving blood pressure target <140/90 mm Hg. *** = *p* value <0.001.

The achievement of HbA1c, LDL‐c, and blood pressure targets is shown in Figure 1. Overall, less than a third of the study population achieved an HbA1c target below 53 mmoL/L (7.0%). In the CVD‐ group, the target was achieved slightly more frequently (*p* = .17). The LDL‐c target was achieved by about half of the study population. Despite lower overall LDL‐c in the CVD+ group, the achievement of this target was significantly lower in the CVD+ group in comparison to the CVD‐ group (35% vs 54%, *p* = .001). Blood pressure targets were achieved by about three quarters of the study population and were achieved more often by individuals in the CVD‐ group (77% vs 45%, *p* < .001).

### 3.2 Characteristics of target achievement in CVD+

Characteristics of individuals with CVD achieving glycemic, LDL‐c, and blood pressure targets were heterogenous, and only a few characteristics differed significantly between those who did or did not achieve targets (Table S1). Individuals achieving glycemic targets were significantly less often smokers and were prescribed less LLM. Those achieving LDL‐c targets had significantly longer diabetes duration, poorer renal function and were prescribed more LLM and AHM. No significant differences were found between those who did or did not achieve blood pressure targets.

**FIGURE 2:**
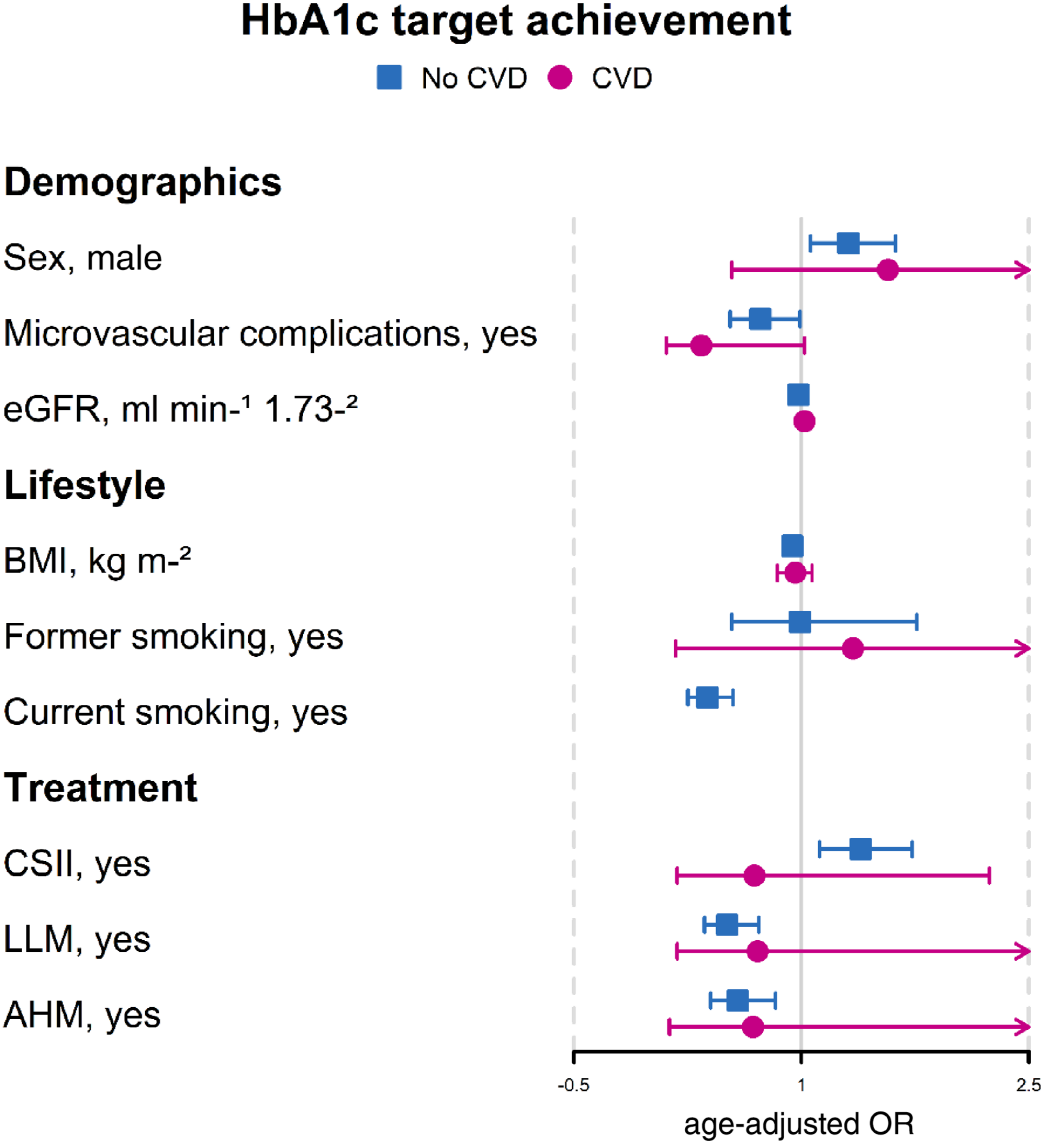
Odds ratios of HbA1c target achievement for those with and without cardiovascular disease, adjusted for age. AHM, antihypertensive medication; BMI, body mass index; CSII, continuous subcutaneous insulin infusion; CVD, cardiovascular disease; eGFR, estimated glomerular filtration rate; HbA1c, glycated hemoglobin; LLM, lipid lowering medication; OR, odds ratio.

**FIGURE 3:**
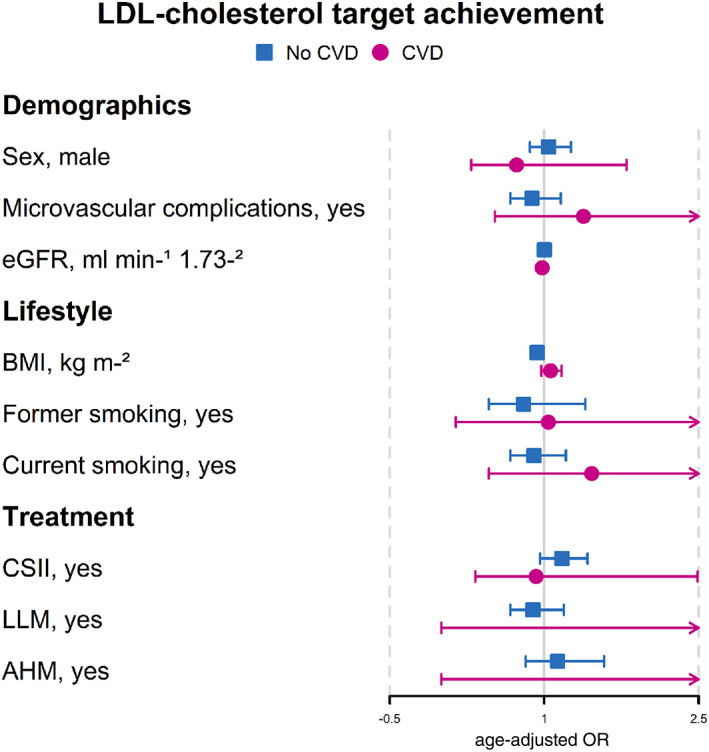
Odds ratios of LDL‐cholesterol (LDL‐c) target achievement for those with and without cardiovascular disease, adjusted for age. AHM, antihypertensive medication; BMI, body mass index; CSII, continuous subcutaneous insulin infusion; CVD, cardiovascular disease; eGFR, estimated glomerular filtration rate; LLM, lipid lowering medication; OR, odds ratio.

**FIGURE 4:**
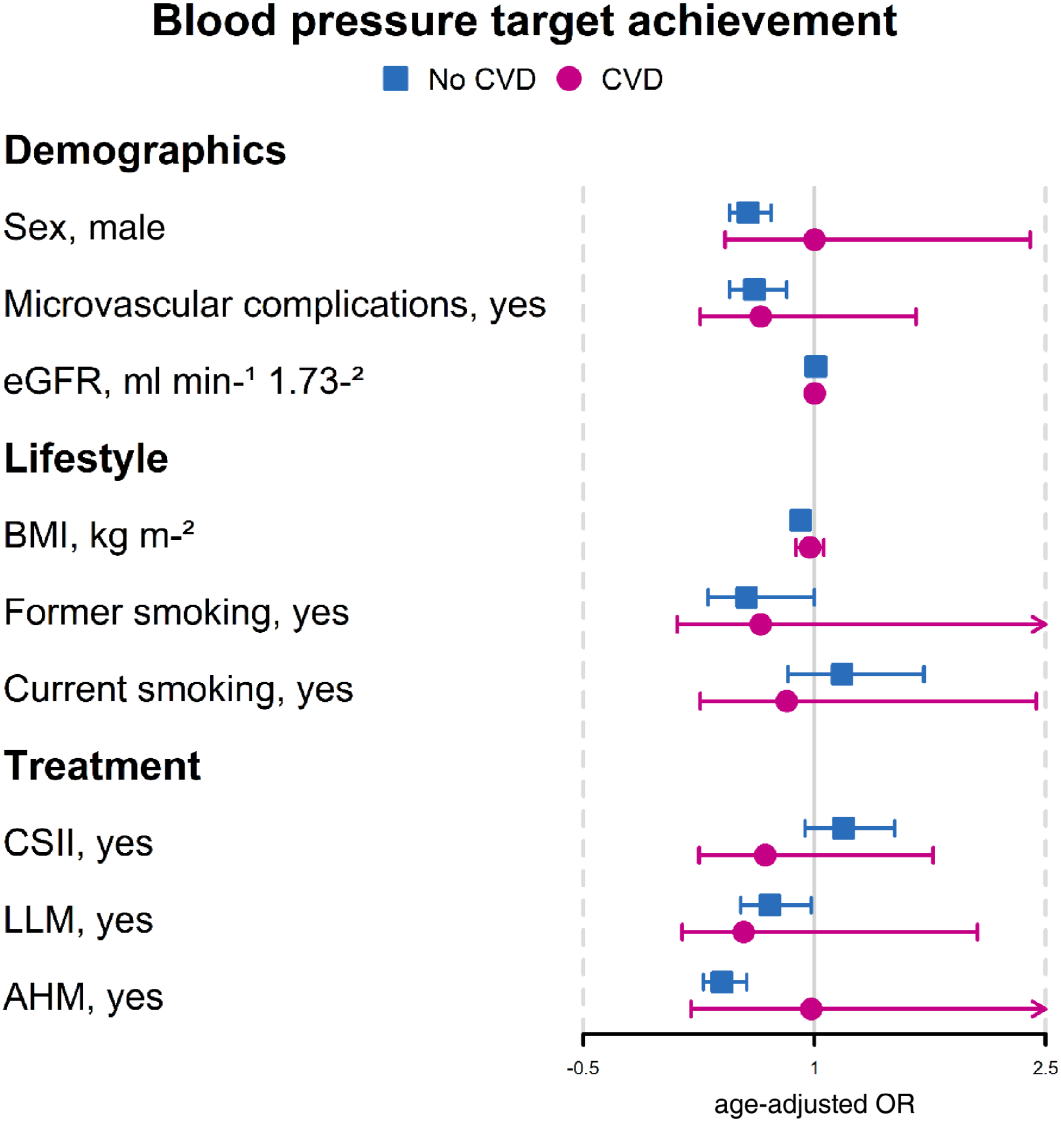
Odds ratios of blood pressure target achievement for those with and without cardiovascular disease, adjusted for age. AHM, antihypertensive medication; BMI, body mass index; CSII, continuous subcutaneous insulin infusion; CVD, cardiovascular disease; eGFR, estimated glomerular filtration rate; LLM, lipid lowering medication; OR, odds ratio.

Figures 2, 3, 4 show the aORs for target achievement for each of the risk factors. Despite some significant differences in characteristics for glycemic and LDL‐c target achievement in individuals with CVD, the aORs showed no significant associations between characteristics and target achievement in this group.

### 3.3 Characteristics of target achievement in CVD−

In contrast, in the CVD− group several factors were found to differ significantly between those who did or did not achieve targets (Table S2). In individuals achieving glycemic targets, individuals were significantly older, more often men, of western European decent, CSII users, more often nonsmokers, with poorer renal function, lower LDL‐c, and less LLM prescribed. BMI and HbA1c were significantly lower in individuals who did or did not achieve LDL‐c targets. Finally, individuals without CVD achieving blood pressure targets were younger, were more often women, had a shorter diabetes duration, were more often CSII users, had lower BMI, less microvascular complications, were more often nonsmokers, had better renal function, and used less LLM and AHM.

When adjusted for age, the association for achieving glycemic targets was significantly greater in men (aOR: 1.31, *p* = .013) and CSII users (aOR: 1.39, *p* = .003) (Figure 2). Those smoking (aOR: 0.38, *p* < .001), with microvascular complications (aOR: 0.73, *p* = .044), and prescribed LLM (aOR: 0.51, *p* = <.001) and AHM (aOR: 0.58, *p* = .004) were significantly less likely to achieve glycemic targets (Figure 2). Other characteristics such as age, diabetes duration, BMI, and renal function were found not to increase chances of glycemic target achievement.

Associations between characteristics and LDL‐c achievement are illustrated in Figure 3. Only diabetes duration and BMI were significantly associated; however, both showed no or very weak associations with LDL‐c achievement.

Finally, men (aOR: 0.57, *p* < .001), individuals with microvascular complications (aOR: 0.61, *p* = .002), and those prescribed AHM (aOR: 0.40, *p* < .001) were significantly less likely to achieve blood pressure targets (Figure 4). Diabetes duration, BMI, and renal function did not show greater odds of blood pressure target achievement.

## 4 DISCUSSION

In this study, we described the achievement of glycemic, LDL‐c, and blood pressure targets in individuals with T1D with and without CVD. Furthermore, we reported the differences between those able to achieve these targets and any associations between characteristics and target achievement.

We found that the majority of our population did not achieve glycemic and LDL‐c targets. In contrast, blood pressure targets were achieved by a great majority. Although suboptimal achievement of glycemic, LDL‐c, and blood pressure targets has been described before in individuals with T1D without CVD,^9^, ^24^, ^25^, ^26^ our study is one of the first to describe target achievement, specifically in individuals with T1D who have established CVD. Individuals with CVD were significantly less likely to achieve LDL‐c targets and blood pressure targets in comparison to those without CVD. As treatment of CVD improves and the life expectancy of individuals with T1D continues to rise, it becomes increasingly important to study ways to improve CVD prevention and target achievement in these individuals.^10^

In individuals with CVD, no measured characteristics were found to be associated with the likelihood to achieve targets. This may suggest that there are other factors, not measured in this study, which are worth investigating. Diet, physical activity, and psychological factors such as stress and anxiety are among the risk factors that could potentially influence target achievement.^27^ However, the small sample size of individuals with CVD may have contributed to the lack of significant associations found between those that did or did not achieve targets. Larger studies in this subgroup of individuals with T1D may be beneficial. As individuals with CVD are significantly less likely to achieve targets, there is great importance in improving management strategies in this group.

Within the subgroup of individuals without CVD, certain characteristics were demonstrated to affect the likelihood of target achievement, which may help to identify individuals who require more attention. Smoking and the prescription of LLM and AHM were negatively associated with glycemic target achievement, suggesting that individuals with known CVD risk factors such as smoking, dyslipidemia, and hypertension are less likely to achieve glycemic targets. Considering the importance of glycemic management for CVD risk reduction, these individuals may be at greater risk for future CVD events.^3^, ^28^ Furthermore, in our study blood pressure targets were less likely to be achieved if an individual was a man, had microvascular complications, and was prescribed AHM.

Individuals who have developed microvascular complications have an even greater risk for developing CVD, particularly individuals with diabetic nephropathy.^28^ As AHM prescription indicates either the presence of hypertension or microalbuminuria, our study further suggests that individuals who fail to achieve targets are those comorbid for established risk factors.^28^ Although these findings illustrate an association between comorbid CVD risk factors and target achievement, further longitudinal studies could shed light on potential causal relationships.

Despite an overall lower LDL‐c in the group with CVD, fewer individuals were able to reach the target of 1.8 mmol/L. Recently, the European Society of Cardiology/European Atherosclerosis Society guidelines have further lowered the LDL‐c target for individuals with CVD to <1.4 mmoL/L.^29^ Whether these targets can be met is ultimately based on the prescription practices of health care providers.^29^ Therapeutic inertia as a result of insufficient training and lack of knowledge of treatment options are some of the barriers for optimal LDL‐c management among health care providers.^30^ Addressing these issues will be necessary in order to improve LDL‐c management.

Lastly, it is important to acknowledge that for many individuals with T1D HbA1c targets are difficult to attain and can lead to a higher frequency of hypoglycemic episodes and, importantly, can become a source of frustration and feelings of failure.^31^ Factors such as fear of hypoglycemia, lack of access to technological devices, and self‐efficacy are just a few areas that can influence glycemic management.^31^ The role of the health care provider is to recognize these potential pitfalls and to help guide those in their care.

### 4.1 Strengths and limitations

Strengths of this study include the large sample size, as well as the use of real‐world data from six diabetes centers in the Netherlands.

This study has three limitations. First, the cross‐sectional study design hampers any conclusion on the causality of the associations found. The presence of CVD risk factors was found to be negatively associated with target achievement. However, it is unclear whether the risk factors themselves were barriers to target achievement or a consequence of it. Nonetheless, this study provides interesting insights and provides some directions for further research. Second, data quality may be limited by the completeness of medical records. In particular, albumin creatinine ratios were unavailable for over >50% of individuals included in this study. However, the missing data rate was comparable to other registry‐based studies. Third, no data were available on the reasons for medication discontinuation. Particularly for LLM, intolerances, patient preferences, or use of alternative supplements such as red rice yeast were not recorded, which could have provided more insights as to why LLM was not prescribed for some individuals despite recommendations.

### 4.2 Recommendations

Further research should be conducted on the temporal relationship between these characteristics and target achievement. In particular, more attention and research are required for the management of individuals with T1D and CVD, as these individuals are the least likely to achieve treatment targets.

## 5 CONCLUSIONS

In conclusion, this study emphasizes that individuals with T1D with established CVD are at a greater risk for not achieving lipid and blood pressure targets. Opportunities for the improvement of glycemic, lipid, and blood pressure management exist but may differ between individuals with and without CVD.

## AUTHOR CONTRIBUTIONS

Rita D. M. Varkevisser contributed to the design, data analysis, and interpretation of the results and authored the paper. Erwin Birnie contributed to the design and interpretation of the results and supervised the work. Dick Mul and Peter R. van Dijk contributed to the design and interpretation of the results. Henk‐Jan Aanstoot, Bruce HR Wolffenbuttel, and Melanie M. van der Klauw supervised the work and, in that role, contributed to the design, data interpretation, and writing of the paper.

## CONFLICT OF INTEREST STATEMENT

Erwin Birnie, Dick Mul, and Henk‐Jan Aanstoot are employed at Diabeter Netherlands, an independent clinic (owned by Medtronic), with brand‐agnostic prescription under EU/Dutch health care laws. The research presented here was independently performed and there are no conflicts of interest.

## Supporting information


**Table S1.** Differences in characteristics between individuals with cardiovascular disease (CVD) achieving glycated hemoglobin (HbA1c), LDL‐cholesterol (LDL‐c), and blood pressure (BP) targets.
**Table S2.** Differences in characteristics between individuals without cardiovascular disease (CVD) achieving glycated hemoglobin (HbA1c), LDL‐cholesterol (LDL‐c), and BP targets.
