# Supplementary material for: Type 1 diabetes management: Room for improvement
Source: J Diabetes. 2023 Feb 17;15(3):255–63. doi: 10.1111/1753-0407.13368 (PMC10036258; doi:10.1111/1753-0407.13368)
Supplement: Supplementary file 1 — Table S1. Differences in characteristics between individuals with cardiovascular disease (CVD) achieving glycated hemoglobin (HbA1c), LDL‐cholesterol (LDL‐c), and blood pressure (BP) targets. Table S2. Differences in characteristics between individuals without cardiovascular disease (CVD) achieving glycated hemoglobin (HbA1c), LDL‐cholesterol (LDL‐c), and BP targets. [file JDB-15-255-s001.docx]

# Supplements

Supplemental Table 1 Differences in characteristics between individuals with cardiovascular disease (CVD) achieving HbA1c, LDL-cholesterol (LDL-c), and blood pressure (BP) targets. Data are presented as means ± SD, medians (quartile 1, quartile 3), and n (%). * p < 0.05, ** p < 0.01, *** p < 0.001.

|  | HbA1c >7%  (n = 69) | HbA1c ≤ 7%  (n= 18) | LDL-c > 1.8 mmol/l  (n= 58) | LDL-c ≤ 1.8 mmol/l  (n = 30) | BP > 140/90 mmHg  (n = 48) | BP ≤ 140/90 mmHg  (n = 39) |
| --- | --- | --- | --- | --- | --- | --- |
| **Demographic and anthropometric measures** | | | | | | |
| **Age, years** | 61 (52, 66) | 62 (57, 68) | 60 (49, 66) | 60 (57, 68) | 62 (54, 68) | 59 (49, 65) |
| **Sex, % women** | 30 (43) | 6 (33) | 25 (39) | 15 (47) | 20 (42) | 16 (41) |
| **Ethnicity, % Western European** | 68 (99) | 18 (100) | 64 (100) | 31 (97) | 47 (98) | 39 (100) |
| **Diabetes duration, years** | 40 (32, 50) | 41 (36, 49) | 37 (26, 46) | 42 (36, 52) * | 40 (34, 53) | 39 (29, 46) |
| **CSII, % yes** | 21 (30) | 4 (22) | 18 (28) | 9 (28) | 15 (31) | 10 (26) |
| **BMI, kg/m2** | 27.3 ± 5.2 | 26.3 ± 4.3 | 26.4 ± 5.5 | 28.2 ± 3.6 | 27.4 ± 5.5 | 26.7 ± 4.5 |
| **Systolic blood pressure, mmHg** | 141 ± 18 | 141 ± 15 | 141 ± 19 | 139 ± 16 | 152 ± 15 | 127 ± 9 |
| **Microvascular complications, % yes** | 50 (72) | 9 (50) | 43 (67) | 23 (72%) | 37 (73) | 28 (64) |
| **Smoking, % yes** |  | * |  |  |  |  |
| Current smoker count, % | 18 (26) | 0 (0) | 13 (21) | 8 (25) | 10 (21) | 8 (21) |
| Former smoker count, % | 5 (7.4) | 2 (11) | 7 (11) | 2 (6) | 4 (8.3) | 3 (7.9) |
| Never smoker count, % | 45 (66) | 16 (89) | 43 (68) | 22 (69) | 34 (71) | 27 (71) |
| **Laboratory measurements** | | | | | | |
| **HbA1c, mmol/mol** | 67 ± 11 | 49 ± 5 | 63 ± 13 | 61 ± 12 | 63 ± 13 | 64 ± 13 |
| **HbA1c, %** | 8.3 ± 1.0 | 6.6 ± 0.4 | 8.0 ± 1.2 | 7.7 ± 1.1 | 7.9 ± 1.1 | 7.9 ± 1.2 |
| **eGFR, ml min¯¹ 1.73¯²** | 61 (50, 79) | 67 (60, 80) | 71 (57, 82) | 57 (50, 72) * | 64 (53, 81) | 62 (53, 77) |
|  |  |  |  |  |  |  |
| **LDL-cholesterol, mmol/L** | 2.34 ± 0.90 | 2.30 ± 0.81 | 2.73 ± 0.79 | 1.52 ± 0.24 | 2.38 ± 0.93 | 2.24 ± 0.87 |
| **Medication** | | | | | | |
| **Lipid lowering medication, % yes** | 61 (88) | 15 (70) * | 49 (77) | 30 (94) * | 43 (89) | 33 (85) |
| **Antihypertensive medication, % yes** | 63 (91) | 18 (88) | 53 (83) | 32 (100) * | 47 (92) | 38 (89) |
| **Antihypertensive medication count** |  |  |  |  |  |  |
| None, % | 7 (10) | 2 (11) | 12 (18) | 0 (0.0) | 5 (10.4) | 4 (10) |
| One, % | 22 (32) | 4 (22) | 19 (30) | 8 (25) | 13 (27) | 13 (33) |
| Two, % | 21 (30) | 6 (33) | 19 (30) | 13 (41) | 16 (33) | 11 (28) |
| Three or more, % | 19 (28) | 6 (33) | 14 (22) | 11 (34) | 14 (29) | 11 (28) |
| **Platelet aggregation inhibitors, % yes** | 37 (55) | 11 (61) | 29 (51) | 19 (53) | 27 (56) | 21 (54) |

Supplemental Table 2 Differences in characteristics between individuals without cardiovascular disease (CVD) achieving HbA1c, LDL-cholesterol (LDL-c), and BP targets. Data are presented as means ± SD, medians (quartile 1, quartile 3), and n (%). * p < 0.05, ** p < 0.01, *** p < 0.001.

|  | HbA1c > 7%  (n = 1172) | HbA1c ≤ 7%  (n = 478) | LDL-c > 2.6 mmol/l  (n = 762) | LDL-c ≤ 2.6 mmol/l  (n = 887) | BP > 140/90 mmHg  (n = 394) | BP ≤ 140/90 mmHg  (n = 1,256) |
| --- | --- | --- | --- | --- | --- | --- |
| **Demographic and anthropometric measures** | | | | | | |
| **Age, years** | 25 (21, 37) | 28 (23, 41) *** | 25 (22, 37) | 26 (22, 40) | 34 (24, 54) | 25 (22, 33) *** |
| **Sex, % women** | 620 (53) | 220 (46) * | 392 (52) | 444 (50) | 160 (40) | 680 (54) *** |
| **Ethnicity, % Western European** | 1,094 (93) | 478 (96) * | 708 (93) | 836 (95) | 374 (95) | 1,179 (94) |
| **Diabetes duration, years** | 15 (10, 21) | 15 (9, 24) | 15 (10, 22) | 15 (10, 22) | 19 (12, 32) | 15 (9, 20) *** |
| **CSII, n % yes** | 610 (52) | 282 (59) * | 398 (53) | 492 (56) | 189 (48) | 703 (56) * |
| **BMI, kg/m2** | 25.8 ± 4.7 | 24.8 ± 3.6 *** | 26.1 ± 4.7 | 24.9 ± 4.1 *** | 27.2 ± 4.9 | 25.0± 4.1 *** |
| **Systolic blood pressure, mmHg** | 131 ± 13 | 130 ± 13 | 131 ± 12 | 130 ± 13 | 147 ± 10 | 125 ± 9 *** |
| **Microvascular complications** | 224 (19) | 86 (18) | 140 (18) | 163 (18) | 128 (33) | 182 (15) *** |
| **Smoking, % yes** |  |  |  |  |  |  |
| Current smoker count, % | 183 (17) | 33 (6.8) *** | 102 (14) | 111 (13) | 48 (13) | 168 (14) *** |
| Former smoker count, % | 35 (3.1) | 19 (4.0) | 24 (3.3) | 28 (3.4) | 26 (7.0) | 28 (2.2) |
| Never smoker count, % | 887 (80) | 407 (89) | 597 (83) | 693 (83) | 300 (80) | 994 (84) |
| **Laboratory measurements** | | | | | | |
| **HbA1c, mmol/mol** | 69 ± 15 | 47 ± 5 | 66 ± 18 | 61± 15 *** | 63 ± 14 | 63 ± 17 |
| **HbA1c, %** | 8.5 ± 1.4 | 6.5 ± 0.4 | 8.1 ± 1.6 | 7.7 ± 1.4 *** | 8.0 ± 1.4 | 7.9 ± 1.5 |
| **eGFR, ml min¯¹ 1.73¯²** | 103 (86, 121) | 93 (79, 110) *** | 100 (84, 120) | 99 (84, 117) | 92 (76, 108) | 103 (87, 120) *** |
|  |  |  |  |  |  |  |
| **LDL-cholesterol, mmol/L** | 2.75 ± 0.81 | 2.54 ± 0.68 *** | 3.34 ± 0.62 | 2.14 ± 0.37 | 2.74 ± 0.80 | 2.68 ± 0.77 |
| **Medication** | | | | | | |
| **Lipid lowering medication, % yes** | 214 (18) | 67 (14) * | 125 (16) | 150 (17) | 115 (29) | 166 (13) *** |
| **Antihypertensive medication, % yes** | 166 (14) | 58 (12) | 89 (12) | 129 (15) | 118 (30) | 106 (8.4) *** |
| **Antihypertensive medication count** |  |  |  |  |  |  |
| None, % | 988 (86) | 411 (88) | 660 (88) | 739 (85) | 270 (70) | 1,129 (92) *** |
| One, % | 114 (9.9) | 38 (8.1) | 61 (8.2) | 91 (10) | 75 (20) | 77 (6.3) |
| Two, % | 38 (3.3) | 14 (3.0) | 25 (3.0) | 27 (2.9) | 29 (7.8) | 23 (1.9) |
| Three or more, % | 14 (1.2) | 5 (1.1) | 5 (0.7) | 14 (1.6) | 14 (3.8) | 5 (0.4) |
| **Platelet aggregation inhibitors, % yes** | 10 (1.2) | 2 (0.4) | 3 (0.4) | 9 (1.0) | 4 (1.0) | 8 (0.6) |
